# Biotinylation of Deoxyguanosine at the Abasic Site in Double-Stranded Oligodeoxynucleotides

**DOI:** 10.1155/2016/4681421

**Published:** 2016-10-12

**Authors:** Chun Wu

**Affiliations:** Biomedical Research Institute, AIST, 1-8-31 Midorigaoka, Ikeda, Osaka 563-8577, Japan

## Abstract

Biotinylation of deoxyguanosine at an abasic site in double-stranded oligodeoxynucleotides was studied. The biotinylation of deoxyguanosine is achieved by copper-catalyzed click reaction after the conjugation of the oligodeoxynucleotide with 2-oxohex-5-ynal. The biotinylation enables visualization of the biotinylated oligodeoxynucleotides by chemiluminescence on a nylon membrane. In order to investigate the biotinylated site, the biotinylated oligodeoxynucleotides were amplified by the DNA polymerase chain reaction. Replacement of guanine opposing the abasic site with adenine generated by the activity of the terminal deoxynucleotidyl transferase of DNA polymerase was detected by DNA sequencing analysis and restriction endonuclease digestion. This study suggests that 2-oxohex-5-ynal may be useful for the detection of the unpaired deoxyguanosine endogenously generated at abasic sites in genomic DNA.

## 1. Introduction

It is well known that glyoxal or its derivatives react with N1 and C2 amino groups of guanine to form a stable three-ring structure [1]. In particular, kethoxal compound, which selectively reacts with guanine, is useful for structural probing studies of the single-stranded RNA aptamers [2]. Another glyoxal derivative, 3′,4′,5′-trimethoxyphenylglyoxal (TMPG), has been recently reported to react with the deoxyguanosine in the single-stranded oligodeoxynucleotide selectively [3]. It is interesting that the conjugated product obtained from the aromatic glyoxal has shown weak chemiluminescence under basic conditions. However, the shortage of the conjugations using those glyoxal derivatives is that the conjugated guanines are difficult to be separated from unreacted portion and to be detected effectively.

**Figure 1:**
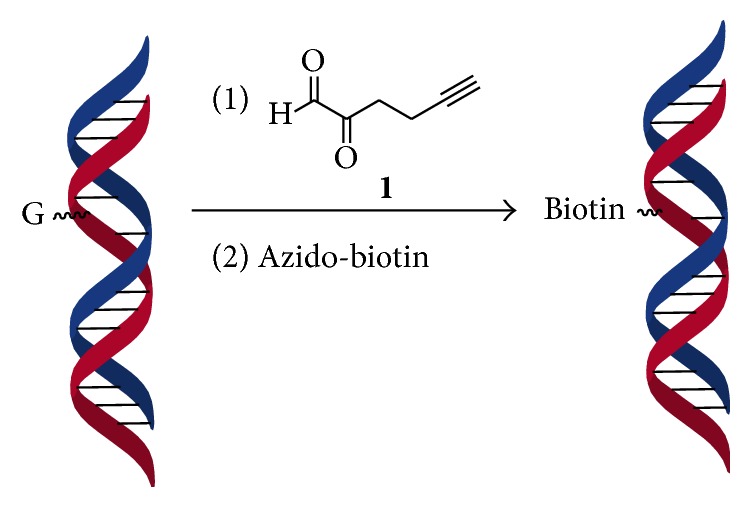
Biotinylation of deoxyguanosine at an abasic site in double-stranded DNA oligonucleotides. The biotinylation of the unpaired deoxyguanosine is achieved by copper-catalyzed click reaction after the conjugation of the oligodeoxynucleotide with 2-oxohex-5-ynal (**1**).

Recently, the reagent 2-oxohex-5-ynal (**1**) has been reported for the biotinylation of the arginine residue in proteins using click chemistry [4]. Since the biotin molecule has the extremely high noncovalent interaction to avidin and streptavidin, this reagent may have a big advantage over conventional glyoxal derivatives for the biotinylation of guanine. Therefore, in this study, the reagent 2-oxohex-5-ynal was studied for the biotinylation of the unpaired deoxyguanosine opposing an abasic site in 100 bp double-stranded oligodeoxynucleotides (Figure 1). The biotinylation of deoxyguanosine is achieved by copper-catalyzed click reaction after the conjugation of the oligodeoxynucleotide with 2-oxohex-5-ynal. This simple approach enabled the visualization of the biotinylated oligodeoxynucleotide by chemiluminescence on a nylon membrane. Furthermore, the biotinylated DNA enabled easy purification on a monomeric avidin column for subsequent analysis.

## 2. Experimental

The reagent 2-oxohex-5-ynal was prepared according to the previous literature [5]. The 100-mer single-stranded DNA oligonucleotides (SS-G), 5′-(CAG TGA AGT TGG CAG ACT GAG CCA GGT CCC ACA GAT GCA GTG ACC GGA GTC ATT GCC AAA CTC TGC AGG AGA GCA AGG GCT GTC TAT AGG TGG CAA GTC A)-3′, were custom-synthesized (GeneDesign, Japan). The complementary DNA oligonucleotides (SS-abasic site) were prepared from the single-stranded 100-mer oligonucleotides DNA with uracil 5′-(TGA CTT GCC ACC TAT AGA CAG CCC TTG CTC TCC TGC AGA GTT TGG CAA TGA CTC UGG TCA CTG CAT CTG TGG GAC CTG GCT CAG TCT GCC AAC TTC ACT G)-3′. The 100-mer single-stranded DNA oligonucleotides (40 pmol) having one uracil base were treated with 10 units of uracil-DNA glycosylase (UDG) in the UDG reaction buffer at 37°C for 1 h. The solution was heated to 90°C for 1 min and then cooled. After the complement DNA oligonucleotides SS-G (40 pmol) was added, the solution was heated to 100°C for 10 minutes. The resultant double-stranded DNA oligonucleotides DS-G/abasic site was purified with NucleoSpin® Gel and PCR Clean-up kit (Takara Bio, Japan). As a control sample, the double-stranded DNA oligonucleotides DS-G/C without an abasic site was prepared by the PCR amplification with a set of primers, FW primer, 5′-(CAG TGA AGT TGG CAG ACT GAG C)-3′, and Rev primer, 5′-(CTG ACT TGC CAC CTA TAG ACA GC)-3′, and the single-stranded DNA oligonucleotides SS-G as the template. The double-stranded DNA oligonucleotides DS-G/C were also purified with NucleoSpin Gel and PCR Clean-up kit.

5 *μ*L of 2-oxohex-5-ynal (0.3 M) in DMSO was added to the solution of the double-stranded DNA oligonucleotides (8 pmol) with or without an abasic site [3]. The solutions were incubated for 30 mins at 37°C. The reaction mixture was purified on G-50 micro column (GE healthcare, USA). A mixture of 30 *μ*L (0.1 M CuBr/0.1 M TBTA 1 : 2 in DMSO/t-BuOH 3 : 1) and 20 *μ*L of Biotin-PEG_3_-Azide solution (Jena Bioscience, USA) was added to 50 *μ*L of the solution of DNA in Tris buffer. The reaction was incubated at 37°C for 2 h. To remove the excess click reagents, the biotinylated DNA was purified on NAP-5 columns (GE Healthcare, USA). The 2-fold serial dilutions of DNA oligonucleotides with or without an abasic site were spotted on a nylon membrane. The DNA samples were visualized with a commercially available chemiluminescent nucleic acid detection kit (Pierce, USA).

In order to remove nonbiotinylated DNA, the reaction solution was loaded on a monomeric avidin column. After washing away unbound DNA, the biotinylated DNA was eluted and concentrated on NucleoSpin Gel and PCR Clean-up kit (Takara Bio, Japan). The purified DNA oligonucleotides were amplified by the DNA polymerase chain reaction with Taq polymerase and the sets of primers. The PCR amplified products were purified with NucleoSpin Gel and PCR Clean-up kit (Takara Bio, Japan). PCR using the single-stranded DNA oligonucleotides (SS-abasic site) as the template was performed using the same primers set. All PCR amplified products were digested with MspI (New England Biolabs, USA) in NEB buffer 4. The reaction solutions were loaded onto agarose gel (2%) along with DNA ladders.

## 3. Results and Discussion

**Figure 2:**
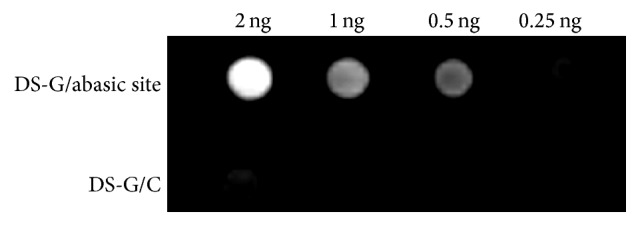
The serial dilutions after biotinylation were spotted on a nylon membrane. The chemiluminescent signals were detected by the chemiluminescent image analyzer.

Conjugation of 2-oxohex-5-ynal with the double-stranded 100-mer DNA oligonucleotides with an abasic site was performed at 37°C according to the previous literature [3]. The obtained samples were biotinylated by the copper-catalyzed azide-alkyne cycloaddition through the acetylene group. For comparison, the double-stranded DNA DS-G/C without an abasic site was treated under same conditions. The serial dilutions were spotted on a nylon membrane and were analyzed by the chemiluminescent detector (Figure 2). The chemiluminescent image indicated that the double-stranded DNA oligonucleotides DS-G/abasic site (upper panel) exhibited considerably higher signal than the double-stranded DNA oligonucleotides G/C (lower panel).

**Figure 3:**
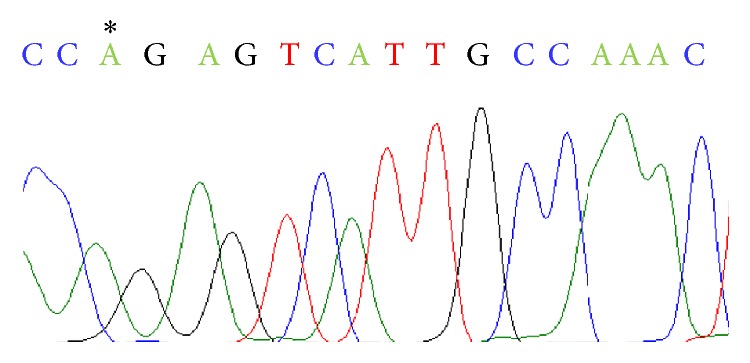
The DNA sequence showed the replacement of guanine to adenine with an asterisk symbol. Replacement of guanine with adenine generated by the activity of the terminal deoxynucleotidyl transferase of Taq polymerase was detected by DNA sequencing analysis.

**Figure 4:**
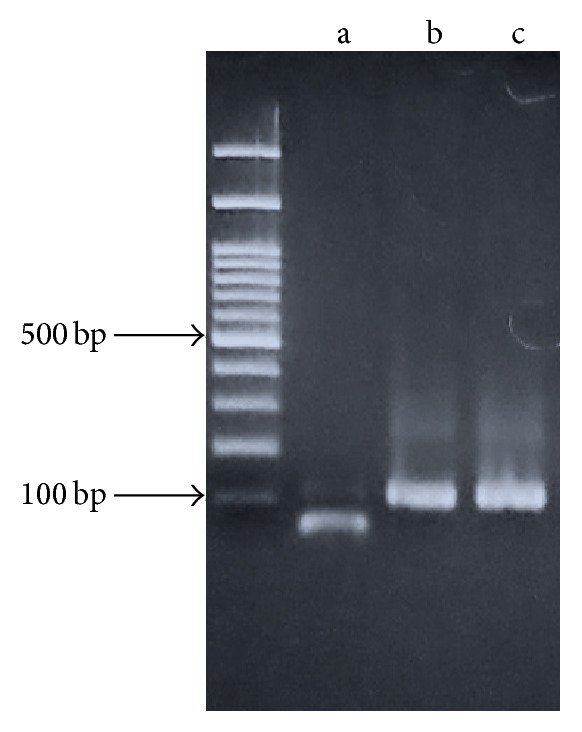
Restriction digestion and gel electrophoresis of the PCR amplified products using SS-G (a), SS-abasic site (b), or the biotinylated DS-G/abasic site (c) as the template. The restriction enzyme MspI which recognizes the CCGG sequence was used to cleave the PCR amplified product.

Next, in order to investigate the biotinylated site in the reaction, the biotinylated DNA was purified on monomeric avidin column and then amplified by PCR chain reaction. To confirm the sequence, the amplified product was examined using the DNA sequencing analysis. As a result, replacement of the base guanine with adenine was observed (Figure 3). No other mutations in the DNA oligonucleotides were observed. The sequence of the PCR amplified product was also examined using a restriction enzyme. The restriction enzyme MspI which recognizes the CCGG sequence was used to cleave the PCR amplified product. For comparison, PCR using the single-stranded DNA oligonucleotides (SS-G or SS-abasic site) as the template was performed. The PCR amplified products were also treated with MspI. Agarose gel electrophoresis revealed that the PCR amplification product using the single-stranded DNA oligonucleotides (SS-G) as the template was clearly cleaved by the restriction enzyme (Figure 4). However, the PCR product amplified from the biotinylated DNA or the single-stranded DNA oligonucleotides (SS-abasic site) as the template was not cleaved by MspI under the same conditions. These observations supported the results of DNA sequencing analysis, in which guanine was replaced with adenine at CCGG sequence of the PCR amplification product. It is well known that Taq DNA polymerase adds preferentially adenine at the blunt end of the amplified product [6]. Once a double-stranded blunt end was formed at the abasic site of the template during the PCR amplification, adenine was inserted by Taq DNA polymerase. In the PCR reaction, using the biotinylated double-stranded DNA oligonucleotides DS-G/abasic site as the template, the single-stranded DNA (SS-G) was blocked by biotinylation and only the complementary DNA having the abasic site could be used as the template. This was why the digestion of the amplified product of the biotinylated double-stranded DNA oligonucleotides was the same as that obtained from the PCR amplified product using the single-stranded DNA oligonucleotides (SS-abasic site) as the template.

In genomic DNA of mammalian cells, cytosine in CpG islands composed of a CG dinucleotide sequence is known to be methylated or demethylated of cytosine. Typically, the presence and absence of methylation in the promoter region of a gene function as an on-off switch for transcription of the gene. Although methylation and demethylation of cytosine have been well studied, the details of demethylation have remained unknown for a long time. However, recently, the mechanism of the active DNA demethylation in mammalian cells has been elucidated [7]. This approach of the demethylation of cytosine is mediated by two enzymes. The ten-eleven translocation (TET) family enzymes oxidized the methyl cytosine and the enzyme thymine DNA glycosylase (TDG) excises the oxidative product. This process generates the unpaired guanine at an abasic site. On the other hand, the bases of the genomic DNA in mammalian cells exposed to chemical substances, ultraviolet ray or X-ray irradiation, or oxidation stress and 50,000~200,000 bases per cell are eliminated at random, creating abasic sites [8]. Therefore, it is important to identify the type of the corresponding base at the abasic sites in DNA. To the best of our knowledge, the unpaired guanine at an abasic site has not been investigated so far. The results from this study suggest that this glyoxal derivative may be useful for the biotinylation of the endogenously generated unpaired guanine at the abasic sites in genomic DNA. Such study using this reagent for real sample is now in progress.
